# SDF-1α Mediates Wound-Promoted Tumor Growth in a Syngeneic Orthotopic Mouse Model of Breast Cancer

**DOI:** 10.1371/journal.pone.0060919

**Published:** 2013-04-11

**Authors:** Christina H. Stuelten, Frances N. Cervoni-Curet, Johanna I. Busch, Emily Sutton, Joshua D. Webster, Sandra L. Kavalukas, Lalage M. Wakefield, Adrian Barbul, John E. Niederhuber

**Affiliations:** 1 Cell and Cancer Biology Branch, National Cancer Institute, Bethesda, Maryland, United States of America; 2 Laboratory of Cellular and Molecular Biology, National Cancer Institute, Bethesda, Maryland, United States of America; 3 Laboratory of Cancer Biology and Genetics, National Cancer Institute, Bethesda, Maryland, United States of America; 4 Sinai Hospital, Department of Surgery, Baltimore, Maryland, United States of America; 5 Johns Hopkins Medical School, Department of Surgery, Baltimore, Maryland, United States of America; Queensland University of Technology, Australia

## Abstract

Increased growth of residual tumors in the proximity of acute surgical wounds has been reported; however, the mechanisms of wound-promoted tumor growth remain unknown. Here, we used a syngeneic, orthotopic mouse model of breast cancer to study mechanisms of wound-promoted tumor growth. Our results demonstrate that exposure of metastatic mouse breast cancer cells (4T1) to SDF-1α, which is increased in wound fluid, results in increased tumor growth. Both, wounding and exposure of 4T1 cells to SDF-1α not only increased tumor growth, but also tumor cell proliferation rate and stromal collagen deposition. Conversely, systemic inhibition of SDF-1α signaling with the small molecule AMD 3100 abolished the effect of wounding, and decreased cell proliferation, collagen deposition, and neoangiogenesis to the levels observed in control animals. Furthermore, using different mouse strains we could demonstrate that the effect of wounding on tumor growth and SDF-1α levels is host dependent and varies between mouse strains. Our results show that wound-promoted tumor growth is mediated by elevated SDF-1α levels and indicate that the effect of acute wounds on tumor growth depends on the predetermined wound response of the host background and its predetermined wound response.

## Introduction

Breast cancer is one of the most frequent malignant tumors observed in American women with a lifetime risk of 12% [1]. Tumor stroma shows an altered histology with increased collagen content, neovascularization and often infiltration by inflammatory cells [2]. Similar stromal alterations are observed during wound healing and scarring. Of concern to surgeons is the potential growth stimulating effect that the host healing response, which follows the surgical removal of a primary tumor, may have on residual cancer cells left in nearby tissues and on the presence of micrometastases.

Experimentally, it has been shown that in Rous sarcoma virus-infected chickens tumor growth will only occur at wounded sites [3]. Likewise, in an orthotopic syngeneic mouse model of breast cancer wounds promote growth of nearby tumors [4]. In both models tumor growth occurred or was promoted in the immediate vicinity of the wound, whereas remote wounds did not accelerate growth of breast tumors. This implies that local changes in the wound microenvironment are of particular importance for wound-promoted tumor growth. Today surgical resection is the most frequently performed procedure in breast cancer treatment, and understanding the mechanisms underlying wound-promoted tumor growth is of particular importance for preventing possible adverse effects of surgery such as local recurrence.

Surgical wounds are acute wounds that are repaired by a predetermined, complex wound healing response that includes inflammation, neovascularization, and matrix deposition and re-organization [5]. During wound healing, cells cross-signal to coordinate the wound healing response by secretion of signaling molecules such as cytokines, chemokines and growth factors [6]. Many of the chemokines and growth factors that are present in wound fluid during wound healing not only attract immune-, stem- or progenitor cells to the wound but also promote cell proliferation, angiogenesis, and collagen deposition. Thus, the local and temporary increase in chemokines and growth factors at the site of surgical tumor excision might define a local microenvironment that supports tumor growth by promoting cell proliferation, angiogenesis, and the deposition of scaffolding matrix.

Stromal derived growth factor-1 (SDF-1 or CXCL12) is a pleiotropic chemotactic cytokine that binds to and signals through a G-protein coupled receptor, CXCR4. SDF-1, which is expressed in two splice variants, SDF-1α and SDF-1ß, regulates cell motility, adhesion, and chemotaxis, as well as proliferation and survival of cells. One of the main functions of SDF-1 in healthy organisms is regulation of trafficking and homing of stem- and progenitor cells and blood vessel formation [7]–[9]. In tumors, SDF-1/CXCR4 signaling has been shown to regulate vascularization of tumors, to foster tumor growth, and to mediate homing of tumor cells to metastatic sites [10], [11].

Here, we used an orthotopic syngeneic mouse model of wound-promoted tumor growth to investigate which effector molecules present in wound fluid confer wound-promoted tumor growth [4]. We identified SDF-1α as a mediator of wound-promoted tumor growth and demonstrated that mouse strains that respond to wounding with elevated SDF-1α levels show a more pronounced increase of tumor growth after wounding than mouse strains that do not exhibit elevated SDF-1α levels after wounding.

## Materials and Methods

### Tissue Culture

4T1 cells were maintained as previously described [4]. For pretreatment with wound fluid, SDF-1α (Peprotech) or AMD3100 (Sigma-Aldrich) cells were grown in DMEM supplemented with 1% wound fluid or 1% plasma, SDF-1α (10 ng/ml) or AMD3100 (10 nM), respectively.

### Mouse Models

All animal studies were approved and in compliance with the National Cancer Institute's Animal Care and Use Committee guidelines. BALB/c female mice (female, age 8–10 weeks,) were purchased from Charles River. Mice of mixed background were generated by breeding BALB/c female mice with C57BL/6JNIcr (Charles River), DBA/2J, AKR/J, or FVB/nJ male mice (The Jackson Laboratory); animals of the F1 generation were used for experiments. To study wound-promoted tumor growth we used an immunocompetent, syngeneic orthotopic mouse model of metastatic breast cancer [4]. Briefly, mice were bilaterally injected with 5000 4T1 cells into the inguinal mammary fat pads. Nine days later animals were wounded by 10 mm full thickness cutaneous incisions in parallel to the inguinal mammary fat pad. In brief, the backs of mice were shaved with clippers, sterilized with 3 scrubs of chlorhexidine followed by a wipe with DPBS or sterile water using sterile gauze pads. A 10 mm full thickness dermal dermal incision parallel to the tumor cell-inocculated mammary fat pad was made for local wounding. Care was taken not to incise the mammary fat pat, which lay immediately under the dermal incision and was exposed during surgery. Wounds were closed by single stitch sutures using Prolene 5–0. All animals undergoing surgery or sham treatment (anesthesia) received gelmeal before returned to their room. Tumor growth was subsequently measured using calipers. The CXCR4 inhibitor AMD3100 (100 ug/100 ul or 5 mg/kg) or DPBS (100 ul, control treatment) was injected i.p. 30 min before wounding or sham treatment, and thereafter once daily. Wound fluid was generated and collected as described previously [4].

### Staining Procedures

Paraffin sections (5 µm) were rehydrated using standard procedures. ***CD34 immunohistochemistry*** **.** Following antigen retrieval (1 mM EDTA pH8, 0.05% Tween) and blocking of nonspecific binding (Vector Laboratories), specimens were incubated with anti-CD34 antibody (1∶200, Abcam). Antigens were visualized by the ABC method/DAB (Vector Laboratories). ***Picrosirus Red.*** Rehydrated sections were incubated in Picrosirius Red solution (0.5 g Direct Red 80 in 250 ml aqueous saturated picric acid, Sigma) for 1 h, and differentiated with 10 mM HCl for 2 min. ***Hematoxylin/Eosin*** staining was performed using standard procedures.

### Cytokine Microarray and ELISA

For cytokine arrays (RayBiotec Mouse Cytokine Array C Series 1000) wound fluids from 3 animals were pooled and analyzed according to the manufacturer’s instructions (RayBiotech). SDF-1α ELISAs were carried out according to the manufacturer’s instructions (R&D Systems).

### Statistical Analysis

Cumulative tumor volume was calculated as previously described [4]. For statistical analysis, the average cumulative tumor volume was calculated for each animal, Gaussian distribution of the data set was assessed using D’Agostino and Pearson omnibus normality test, and Mann-Whitney Test with Dunn’s Multiple Comparison, ANOVA with Dunnet’s Multiple Comparison Test with Bonferroni Multiple Comparison Test or t-test were carried out as appropriate in GraphPad Prism 5.0c. Data are presented as mean ± standard error of mean (SEM) or as mean ±95% confidence interval (CI) as indicated.

## Results

### SDF-1α Levels are Increased in Wound Fluid

**Figure 1:**
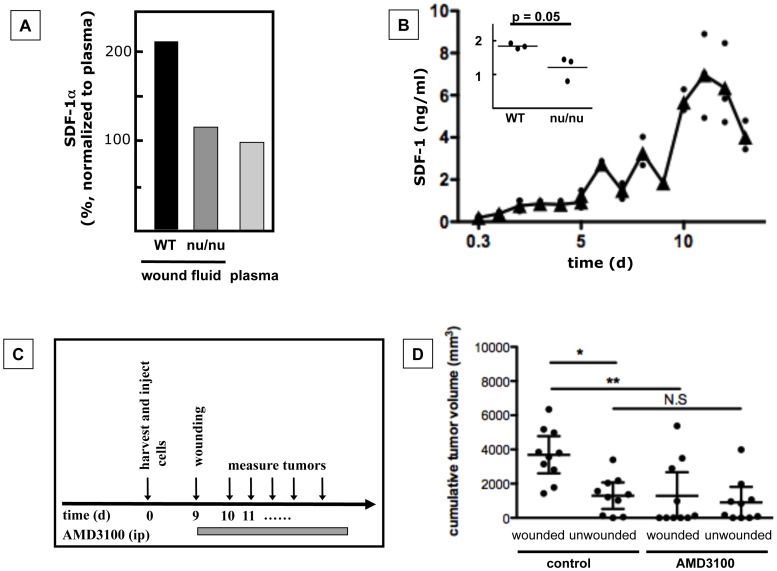
SDF-1α is elevated in wound fluid and increases tumor growth. **A.** SDF-1α levels in wound fluid from BALB/c wildtype (WT) mice were higher than in wound fluid from BALB/c nu/nu mice (nu/nu) or plasma from BALB/c WT animals (cytokine microarray). **B.** SDF-1α levels in wound fluid from BALB/c WT animals increased during the course of wound healing and were higher in WT wound fluid than in wound fluid from nu/nu animals 9d after wounding (insert). n = 5 samples/time point (0.3d to 5d) and n = 3 samples/time point (5d to 14d); Triangle: mean; filled circle: individual data point. Insert: p = 0.05, n = 3, Mann Whitney test). **C, D.** Inhibition of SDF-1α/CXCR4 signaling by AMD3100 treatment abolished wound-promoted-tumor growth. **C.** Experimental design. **D.** Cumulative tumor volumes. p = 0.0027, n = 10 animals/group, Kruskal Wallis Test/Dunn’s Multiple Comparison Test, observation time: 21d, mean ±95% CI.

We have previously demonstrated that wounding increases tumor growth in BALB/c wild type mice, but not in BALB/c nu/nu mice [4]. Furthermore, wound fluid derived from BALB/c wild type mice 9 days after wounding promoted tumor growth when injected in the proximity of tumors [4], indicating that wound fluid contains soluble effector molecules that mediate the effect of wounding on tumor growth. Thus, we hypothesized that wound fluid derived from BALB/c wildtype mice, when compared to wound fluid derived from BALB/c nu/nu mice or plasma, contains increased levels of an effector molecule such as a cytokine or growth factor that promotes tumor growth. Screening of cytokines in wound fluids and plasma by cytokine array demonstrated that SDF-1α levels in wound fluid derived from BALB/c wildtype mice 9 days after wounding are higher than in wound fluid from BALB/c nu/nu mice or plasma (Fig. 1A). Quantification showed that SDF-1α levels in wound fluid of BALB/c mice increased after wounding over 12 days to 7 ng/ml (day12) and then dropped slightly (Fig. 1B). Nine days after wounding, SDF-1α levels were ∼1.5-fold higher in wound fluid derived from wildtype mice than in wound fluid derived from BALB/c nu/nu mice (Fig. 1B, insert) or plasma, confirming the results obtained by cytokine microarray (Fig. 1A).

To confirm that SDF-1α/CXCR4 signaling during wound healing affects tumor growth, we treated wounded or unwounded animals with a small molecule inhibitor of CXCR4, AMD3100, or the carrier DPBS from the day of wounding until euthanasia (Fig. 1C). Treatment of animals with AMD3100 abolished the effect of wounds on tumor growth (Fig. 1D), demonstrating that intact SDF-1α/CXCR4 signaling is necessary for wound-promoted tumor growth.

### SDF-1α Increases Tumor Growth by Directly Affecting Tumor Cells

**Figure 2:**
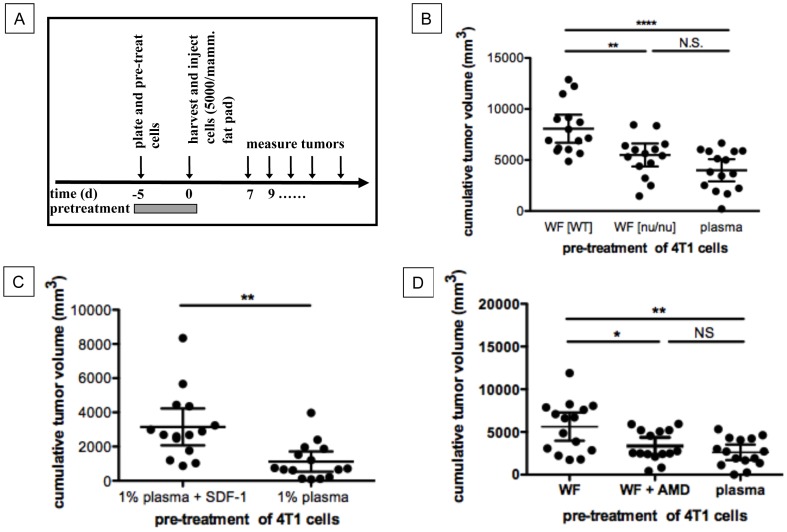
SDF-1α increases tumor growth by directly acting on tumor cells. **A.** Experimental design. **B.** Pretreatment of 4T1 cells with wound fluid derived from WT animals but not wound fluid derived from BALB/c nu/nu animals increased tumor growth *in vivo* as compared to pretreatment of 4T1 cells with plasma. mean ±95% CI, p<0.0001, n = 15 animals/group, ANOVA/Bonferroni’s Multiple Comparison Test, observation time: 18d. **C.** Pre-treatment of 4T1 cells with SDF-1α and plasma *in vitro* resulted in increased tumor growth *in vivo* as compared to pretreatment of 4T1 cells with plasma only. p = 0.0015, n = 15 animals/group, unpaired t-test, observation time: 22d, mean ±95% CI. **D.** Inhibition of SDF-1α/CXCR4 signaling with AMD3100 (AMD) during pre-treatment of 4T1 cells with wound fluid abolished increased tumor growth observed after pretreatment of 4T1 cells with wound fluid. p = 0.0016, n = 15 animals/group, ANOVA/Bonferroni’s Multiple Comparison Test, observation time: 22d, mean ±95% CI.

Wound derived SDF-1α could support tumor growth by a direct effect on tumor cells or indirectly by changing the microenvironment to more actively support tumor growth. In order to distinguish between these two possibilities, we first pretreated 4T1 cells *ex vivo* for 5 days with 1% wound fluid derived from wildtype animals, nu/nu animals, or with 1% plasma, and then trypsinized, washed and orthotopically injected the suspended tumor cells bilaterally into the inguinal mammary fat pads of mice (Fig. 2A). We found that *in vitro* pre-treatment of 4T1 cells with wound fluid derived from wildtype mice significantly increased *in vivo* tumor growth as compared to pre-treatment of 4T1 cells with wound fluid derived from BALB/c nu/nu mice or pre-treatment of 4T1 cells with mouse plasma (Fig. 2B), indicating that an effector molecule in wound fluid derived from wildtype animals directly influences the growth of 4T1 breast cancer cells to form larger tumors *in vivo*.

To further confirm that SDF-1α mediates the effect of wound fluid on tumor cells, we pre-treated 4T1 cells with mouse plasma or mouse plasma and SDF-1α (10 ng/ml) before bilateral orthotopic injection into the inguinal mammary fat pads of BALB/c mice and scored for tumor growth. Pre-treatment of 4T1 cells with SDF-1α correlated with significantly increased tumor growth *in vivo* (Fig. 2C). Furthermore, inhibition of CXCR4-signaling by AMD3100 during pre-treatment of tumor cells with wound fluid abolished the effect of wound fluid on tumor growth (Fig. 2D). This indicates that wound fluid derived SDF-1α mediates wound-promoted tumor growth by stimulating CXCR4-signaling in tumor cells.

### Wound-induced SDF-1α/CXCR4 Signaling Alters Tumor Cell Proliferation, Stromal Composition and Vascularization of Tumors

We next investigated whether the effect of SDF-1α on tumor growth could be explained solely by increased tumor cell proliferation, or if indirect effects such as subsequent stimulation of angiogenesis or matrix deposition contributed to the increased tumor volume after wounding or stimulation of tumor cells with wound fluid or SDF-1α.

**Figure 3:**
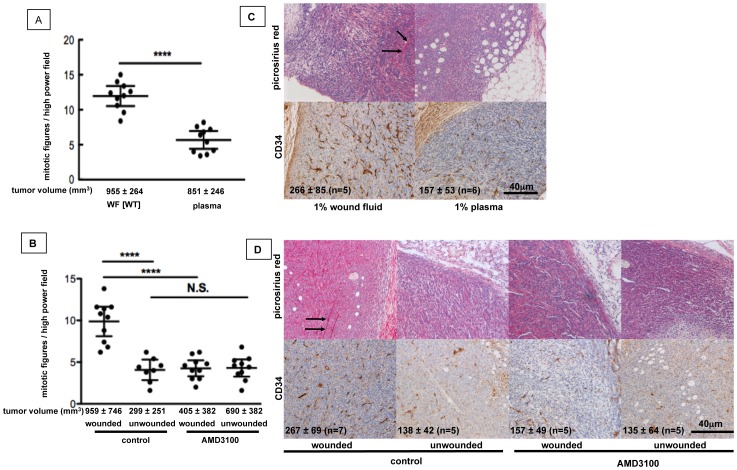
Wound-induced SDF-1α/CXCR4 signaling in tumor cells alters tumor cell proliferation, stromal composition and vascularization of tumors. **A, C**. BALB/c mice were inoculated with 4T1 cells that were pre-treated for 5d with wound fluid or mouse plasma. **B, D.** BALB/c mice were inoculated with 4T1 cells and underwent wounding or sham surgery 9 days later. CXCR4 signaling was systemically inhibited by AMD3100. **A, B.** Mitotic figures in tumor sections. **A.** Unpaired t-test, p<0.0001, n = 10 specimens/group, observation time: 18 days. **B**. Bonferroni’s Multiple Comparison test, p<0.0001, n = 8–12 specimens/group, observation time: 28, mean ±95% CI. **C, D**. Top: Collagen staining with Picrosirius red. Bottom: CD34-positive blood vessels in tumors. Numbers in the lower left corner of images represent the density of CD34-positive structures/mm^3^. **C.** p = 0.0173, Mann-Whitney test. **D.** p = 0.0155, ANOVA, mean ±95% CI).

We found a 2-fold increase in the number of mitotic figures in tumors derived from 4T1 cells that were pre-treated with wound fluid compared to tumors derived from cells that were pre-treated with plasma (Fig. 3A). In contrast, mitotic figures were reduced to half in tumors from wounded AMD3100-treated animals as compared to wounded control animals (Fig. 3B), indicating that wound derived SDF-1α/CXCR4 signaling increases proliferation of 4T1 cells *in vivo*.

Next, we analyzed collagen deposition and density of blood vessels in tumors. Collagen deposition was increased in tumors derived from wound fluid treated cells as compared to tumors derived from plasma treated cells (Fig. 3C), and in tumors from wounded animals as compared to tumors from unwounded animals (Fig. 3D) as shown by Picrosirius Red staining. This effect was abolished by treatment of wounded animals with the CXCR4 inhibitor AMD3100 (Fig. 3D), indicating that wound induced SDF-1α/CXCR4 signaling contributes to collagen deposition in tumors. Similarly, the density of CD34-positive blood vessels was higher in tumors derived from wound fluid treated cells as compared to tumors derived from plasma treated cells (Fig. 3C), and in tumors from wounded animals as compared to tumors from unwounded animals (Fig. 3D) and was decreased to levels seen in control animals by blocking SDF-1α signaling with AMD3100 (Fig. 3D). Taken together, our findings show that wound-derived SDF-1α increases tumor volume by at least three mechanisms: increased tumor cell proliferation, increased collagen deposition, and increased neoangiogenesis. Furthermore, our data imply that these effects can already be elicited by exposure of tumor cells to SDF-1α, indicating that SDF-1α stimulated tumor cells subvert the local microenvironment to support tumor growth.

### Wound-induced SDF-1α Elevation and Subsequent Elevated Tumor Growth is Influenced by the Host Background

**Figure 4:**
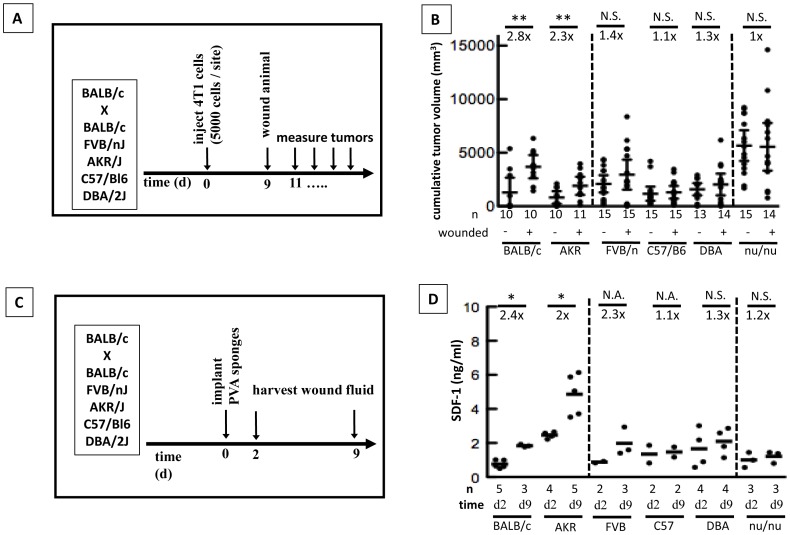
Wound-promoted tumor growth and SDF-1α levels in wound fluid are dependent on the host background. **A,B.** The host background influenced wound-promoted tumor growth. **A.** Wound-promoted tumor growth was assessed in female animals of the F1 generation derived from BALB/c mice bred with BALB/c (control), FVB/nJ, AKR/J, C57Bl/6JNcr, DBA/2J animals. **B**. Cumulative tumor volumes. Unpaired t-test. Mean ±95% CI. Representative of 2 independent experiments is shown. **C,D.** The host background influences SDF-1α levels in wound fluid. **C.** SDF-1α levels were analyzed by ELISA in healthy hosts 2 d or 9d after subcutaneous implantation of PVA sponges. **D.** SDF-1α levels in wound fluid (ELISA). Mann-Whitney test. N.A. : sample number not sufficient to perform statistical analysis. Bar: mean.

The wound healing response following injury can vary greatly between individuals as is evidenced by differences in scar thickness, wound breaking strength, and speed of wound healing. Having demonstrated that wound-derived SDF-1α can affect tumor growth we set out to determine if this response was dependent on the genetic background of the host. We bred female BALB/c animals with male FVB/n, C57/Bl6, DBA, or AKR animals and investigated the effect of wounds on tumor growth using our standard model (Fig. 4A, B). Interestingly, wounding significantly increased tumor growth in BALB/c and BALB/c×AKR animals. BALB/c×FVB/n animals also exhibited increased tumor growth in response to wounding, although this did not reach significance. However, in BALB/c×C57Bl/6 and BALB/c×DBA animals wounding did not affect tumor growth (Fig. 4B). As previously described [4], wounding did not increase tumor growth in BALB/c nu/nu (Fig. 4B).

Having shown that the response of tumors to nearby wounds depends on the host stroma, we next analyzed SDF-1α levels in wound fluid of non-tumor bearing mice 2 days and 9 days after wounding (Fig. 4C, D). Remarkably, BALB/c mice and BALB/c×AKR mice showed wound-promoted tumor growth, and levels of SDF-1α increased 2-fold or more between day 2 and 9 after wounding, while strains that did not show wound-promoted tumor growth (BALB/c×C57Bl/6, BALB/c×DBA, and BALB/c nu/nu) only had a 1.1- to 1.3-fold increase in SDF-1α levels (Fig. 4D). Cumulative tumor volumes were 1.4-fold higher in wounded than in unwounded BALB/c×FVB/n animals in two independent experiments, but this increase of tumor volume did not reach statistical significance (Fig. 4B). Average SDF-1α levels in wound fluid increased 2.3fold after wounding in BALB/c×FVB/n animals, however, due to the low sample number this difference between SDF-1α levels 2d and 9d after wounding could not be assessed statistically (Fig. 4D). Taken together, our data suggest that increased SDF-1α/CXCR4 signaling is an important contributor to wound-promoted tumor growth of 4T1-derived mammary tumors in mice, and that an increase of SDF-1α level in response to wounding may represent a predictive marker of post-surgical growth of residual tumor tissue in the proximity of the wound.

## Discussion

It has been shown that the wound microenvironment can increase tumor take and accelerate tumor growth in animal models as well as increase growth of nearby tumors [4], [12], [13]. We found that the CXCR4 inhibitor AMD 3100 reduces wound-promoted tumor growth without significantly affecting tumor growth in unwounded animals, strongly suggesting that wound-derived SDF-1α, one of the splice variants of SDF-1, increases tumor growth. SDF-1 is overexpressed in cancer-associated fibroblasts of breast tumors and is considered a regulator of tumor stromal interactions [10]. The tumor promoting effect of SDF-1/CXCR4 signaling has been well documented for a variety of epithelial and hematopoietic malignancies and inhibition of CXCR4 signaling has been shown to reduce tumor growth and metastasis [14]. The effects of SDF-1 are complex as it can influence tumor growth at several levels such as survival and growth of cells, cell adhesion, attraction of endothelial progenitors and CXCR4 expressing fibrocytes, as well as altered angiogenesis and matrix formation. [15]–[17].

Here, the tumor-promoting effect of wound-derived SDF-1α was decreased by AMD3100. We observed a multifaceted effect of SDF-1α and AMD3100 on tumor growth: tumor cell proliferation, collagen deposition, and neoangiogenesis were increased by SDF-1α and decreased by AMD3100 regardless whether cells were treated with SDF-1α or AMD3100 *in vitro* or exposed to wound-derived SDF-1α or AMD3100 *in vivo*. Interestingly, while we demonstrate increased tumor cell proliferation *in vivo, in vitro* tumor cell proliferation was only weakly and inconsistently affected by SDF-1α (data not shown). This indicates that SDF-1α may influence proliferation of tumor cells indirectly, for example by stimulating the cells to secrete cytokines, growth factors, or matrix metalloproteinases that then contribute to the orchestration of stromal remodeling and to subsequently increased tumor growth. Likewise, we found increased collagen deposition and neoangiogenesis at the tumor edge in tumors derived from cells treated with SDF-1α *in vitro*, again indicating that SDF-1α stimulates tumor cells to subsequently influence their microenvironment to facilitate tumor growth.

Similarly to the work presented here, AMD3100 reduced wound-promoted tumor growth in a murine model of gastric cancer [18], likely by interfering with recruitment of bone marrow derived stem cells to the tumor and therefore reducing neoangiogenesis. In this gastric cancer study, tumor cells were inoculated on the back of animals while surgery was performed on the abdomen, implying that wounding exerted a systemic effect on tumor growth. In the breast cancer model used here, wound-promoted tumor growth is only observed when wounding occurred in the proximity of the tumor inoculation site [4]. This difference may be due to differences in the animal models used, such as different mouse strains, tumor cell lines, and/or the extent of the surgical trauma. In both models, SDF-1/CXCR4 signaling was implicated as a mediator of wound-promoted tumor growth, demonstrating that this signaling pathway can mediate adverse effects of surgery on tumor growth.

Having shown that SDF-1α/CXCR4 signaling is involved in wound-promoted tumor growth in BALB/c animals, but not in BALB/c nu/nu animals, we wondered how wound-promoted tumor growth varied between strains and if and SDF-1α levels would shift concordantly. We therefore measured SDF-1α levels in wound fluid of different mouse strains and in a second set of animals analyzed wound-promoted tumor growth. In agreement with the proposed role of SDF-1α as one regulator of wound-promoted tumor growth we found that wounds promoted growth of nearby tumors in strains that had elevated SDF-1α levels after surgery, while mouse strains that did not show wound-promoted tumor growth did not show elevated levels of SDF-1α after surgery. However, other factors might influence wound-promoted tumor growth and modulate the effect of SDF-1α on wound-promoted tumor growth, and might be especially important if tumor cells do not express SDF-1 receptors. Together, we envision that SDF-1α levels in wound fluid may be an accessible and easily measurable parameter that could be utilized to predict a potential adverse response of patients to tumor surgery. Furthermore, our results imply that peri- and postoperative inhibition of SDF-1α/CXCR4 signaling may reduce the risk of increased tumor growth in patients that have increased SDF-1α levels after wounding and have incompletely resected CXCR4 positive tumors. Of relevance in the context of a potential clinical application of AMD3100, although we did not perform specific experiments to assess wound healing, we did not observe major effects of ADM3100 on wound healing in treated animals, nor did we observe impaired wound healing or wound stability (data not shown). AMD3100 administration has been described to improve ischemia-mediated tissue damage and closure of excisional skin wounds in diabetic mice [19], [20] but also has been shown to reduce collagen expression and to subsequently impact bone healing [21]. Clinically, AMD3100 (Plerixafor) is approved and has been used for treatment of HIV and mobilization of bone marrow stem cells. Impaired wound healing is not listed as a major side effect [22] implying that peri- and postoperative treatment of breast cancer patients to prevent wound-promoted growth of residual breast tumor tissue might be feasible. Future studies will thus evaluate the role of SDF-1α in wound-promoted tumor growth in the context of clinical settings.
